# Supplementary material for: Chromothripsis during telomere crisis is independent of NHEJ, and consistent with a replicative origin
Source: Genome Res. 2019 May;29(5):737–49. doi: 10.1101/gr.240705.118 (PMC6499312; doi:10.1101/gr.240705.118)
Supplement: Supplemental Material [file supp_gr.240705.118_Supplemental_file_1.zip › contigs/annotated_contigs/DB57/contig.2.DB57_length_545_mean_cov_5.50458715596.docx]

**DB57_length_545_mean_cov_5.50458715596**

```
CCTGTAATCCTAGCATTTTGGGAGCCGAGGTGGGTGGATCATCTGAGGTCAGGGGTTCGAGACCAGCCTGGCCAACATGGCAAAACCCT
 >chr7:134937754-134937964 - E=1e-111
GTCTCTACTAAAAATACAAAAATTAGCTGAGCATAGTGGCATGCACCTGTAATCCCAGCCACTCGGCAGGCTGAGGCAGGAGAATCACT

TGAACCCAGGAGGCGGAGGTTGCAGTGAGC|CA|CATGGGGATTATAATTCAACAGGAGATTTGGGTGGGGACACAGAGCCAAATCAGA
                               >chr7:134937163-134937500 + E=3e-191
TCTGGTGCCTAATTCAGTGGGTATTCTTGGGGATCCACCTTTTTTCTGTGATTGGAAGTGAGCTGGTCCTAGGATTGTGTCATTGCCCT

GGGGATGACACTATCTGTGGCATCTTCAGAGTAGAAAATTCTTCCATCCTGGCGCAGGGCTGGGACATACCAGGTTATCAGCTGTAGGT

TGTGGAAACTCATCTGTTCACTTTGCACCCCATTCCCAGTGTGTGTTAGTCCTGCTAACCTCCACTGAGGGCTGCTGTATCCCTGCCTT

AAGTCCATTATAT
```
